# Low expression of adenomatous polyposis coli 2 correlates with aggressive features and poor prognosis in colorectal cancer

**DOI:** 10.1080/21655979.2020.1820823

**Published:** 2020-09-20

**Authors:** Yan Sun, Hua Tian, Xuehu Xu, Lin Wang

**Affiliations:** a Department of Gastroenterology, The Third Affiliated Hospital of Guangzhou Medical University, Guangzhou, China; b Department of Gastroenterology, Houjie Hospital Affiliated to Guangdong Medical College, Dongguan, China; c Department of General Surgery, The Third Affiliated Hospital of Guangzhou Medical University, Guangzhou, China; d Department of Oncology, Guangzhou Red Cross Hospital, Medical College, Jinan University, Guangzhou, China

**Keywords:** Adenomatous polyposis coli 2, colorectal cancer, tissue microarray, prognosis

## Abstract

Currently, there are no relevant findings on the diagnostic and prognostic roles of adenomatous polyposis coli 2 (APC2) in colorectal cancer (CRC). This study investigated the clinical value of APC2 dysregulation in the prognosis of CRC. Immunohistochemical scores obtained from tissue microarrays were used to quantify the expression of APC2 protein in 201 CRC tissues and 139 adjacent normal tissues. A chi-squared test was performed to analyze the association between APC2 expression and various clinical characteristics. Differences in 5-year survival between groups were analyzed. A receiver operating characteristic (ROC) curve was generated to investigate the potential association between APC2 and CRC diagnosis. Compared with adjacent normal tissues, APC2 was downregulated in CRC tissues (P = 0.0004). Survival analyses revealed that CRC patients with high APC2 expression (96.74%) obtained better 5-year survival rates than those with low APC2 expression (88.07%). CRC patients with low APC2 expression exhibited obvious lymphovascular invasion (P = 0.010), lymph node metastasis (P = 0.007), and high tumor node metastasis (TNM) stage (P = 0.007). Furthermore, ROC curves confirmed that APC2 was associated with lymphovascular invasion (P = 0.004), lymph node metastasis (P = 0.002), and TNM staging (P = 0.002). In summary, low APC2 expression in CRC tissues was associated with poor prognosis and may be a useful biomarker for the prognosis and clinical classification of CRC.

## Graphical abstact

**Figure:**
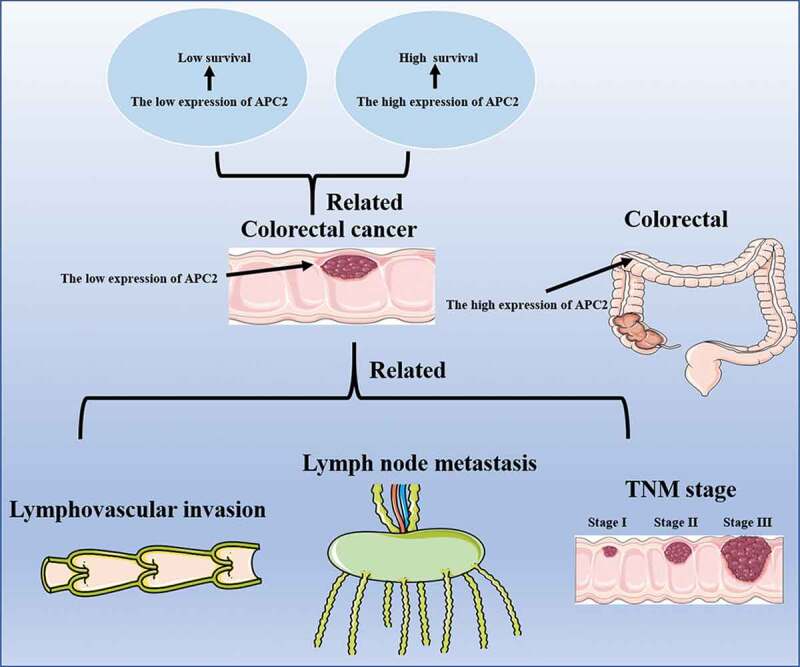

## 1. Introduction

Colorectal cancer (CRC) is one of the most common causes of cancer-related deaths in the world [1]. By 2035, due to population growth and aging, the death toll from colon and rectal cancers in all countries is expected to increase by 60.0% and 71.5%, respectively [2]. This represents a significant burden to the development of the global economy and poses a challenge to CRC control. Studies focusing on the contribution of CRC-related molecules will provide important clues to improve CRC diagnosis, treatment, and prevention [3]. Currently, the molecular factors involved in the etiology of CRC have not been fully elucidated; however, the molecular and functional characteristics of most relevant forkhead box molecules have been associated with CRC susceptibility and prognosis [4].

Recently, studies have suggested that mutations in the adenomatous polyposis coli (APC) gene are associated with CRC [5]. The APC2 gene is homologous to APC and exhibits overlapping functions with APC [6]. Silencing APC2 attenuates the inhibitory effects on cell proliferation and the Wnt signaling pathway in cancer cells, in which G9a is silenced or suppressed [7]. APC2 has been demonstrated to play an important role in regulating neurodevelopment and is one of the important genes that causes Sotos syndrome [8]. Furthermore, APC2 is associated with tumor progression. Inhibition of APC2 expression has been shown to inhibit proliferation of lung cancer cells [9]. In addition, APC2 overexpression suppresses cell proliferation and migration ability in osteosarcoma cells [10]. It has also been reported that methylation-related downregulation of APC2 may be involved in regulating chemotherapy response in tongue squamous cell carcinoma and acute myeloid leukemia patients [11,12].

Increasing evidence has implicated a role of APC in CRC. Early studies have shown that APC mutations promote the growth of early colorectal adenomas [13]. APC mutations may modulate diverse molecular targeted therapies in CRC patients [14]. However, there have been limited studies of APC2 expression in the diagnosis and prognosis of CRC patients. Here, tissue microarrays were performed to analyze the expression of APC2 protein in CRC tissues. A chi-squared test was performed to analyze the association between APC2 expression and various clinical characteristics of CRC. Furthermore, we generated a receiver operating characteristic (ROC) curve to investigate the potential association between APC2 and CRC diagnosis, evaluating the biomarker potential of APC2 in CRC diagnosis and prognosis.

## 2. Materials and methods

### 2.1. Patients and specimens

A total of 201 CRC patients underwent tumor resection at the Third Affiliated Hospital of Guangzhou Medical University, the Houjie Hospital Affiliated to Guangdong Medical College and the Guangzhou Red Cross Hospital, Medical College, Jinan University. Adjacent normal tissue was also collected as a control. Tissues were washed with phosphate buffered saline, fixed in formalin, and paraffin-embedded to prepare pathological sections. The study patients did not receive adjuvant chemotherapy or radiotherapy prior to surgery. General demographic data, clinical pathological diagnosis, staging, and survival times were obtained from the patient’s admission information. The diagnosis of CRC was based on the results of pathological examination by the researchers. Tumor node metastasis (TNM) classification was based on the 7th edition of the TNM classification guide [15]. Informed consent was obtained from each patient and the study was approved by the institutional review board of each of the above participating institutions.

### 2.2. Tissue microarray and immunohistochemistry (IHC)

Tissue preparation was done according to methods described previously [16]. Briefly, 3–4 mm thick tissues were fixed with 4% buffered formalin, paraffin-embedded, and stained with hematoxylin and eosin (H&E; Solarbio, Beijing, China). For the H&E protocol, representative tissue blocks were cut into 0.8-mm sections for tissue microarray construction. Using specific instruments for the tissue microarray (Beecher Instruments, Sun Prairie, WI, USA), approximately 5-μm sections were cut to generate tissue microarray slides for APC2 expression analysis. The tissue microarray procedure was performed by Guangzhou Wanshan Biotechnology Co., Ltd (Guangzhou, China). Three cores were arrayed per specimen. APC2 expression was assessed based on the percentage of cells expressing APC2 and the staining intensity. The percentage (P) of cells expressing APC2 was obtained using Image Pro Plus 6.0 software. The staining intensity (I) was evaluated by two blinded researchers and based on a scale (0–3) as follows: 0, negative staining; 1, weak staining; 2, medium staining; 3, strong staining. The APC2 staining level was assessed by the immunoreactive score (IRS) from the P and I of the APC2-positive cells: IRS = P × I [17]. IRS = 0 represented negative and IRS > 0 represented positive expression.

### 2.3. Survival and ROC analysis

CRC patients were divided into two groups: APC2-high expression and APC2-low expression. The high expression group included the patients with expression of APC2 protein higher than that of the median value, based on all CRC patient measurements, whereas the low expression group was defined as those exhibiting lower expression than the median value. The 5-year survival time of CRC patients was also determined. To investigate the diagnostic value of APC2 in CRC, ROC curves were generated.

### 2.4. Statistics

All the data were analyzed using SPSS 18.0 version software (SPSS Inc., Chicago, IL, USA). Univariate analysis was performed to investigate the association between the expression of APC2 and clinicopathological characteristics of CRC patients with the chi-squared test. For prognostic analysis of APC2 in CRC, survival curves were generated using the Kaplan–Meier method, and the differences in 5-year survival time were analyzed by the log-rank method. A p-value of <0.05 was considered statistically significant.

## 3. Results

### 3.1. APC2 is downregulated in colorectal cancer

**Figure 1.**
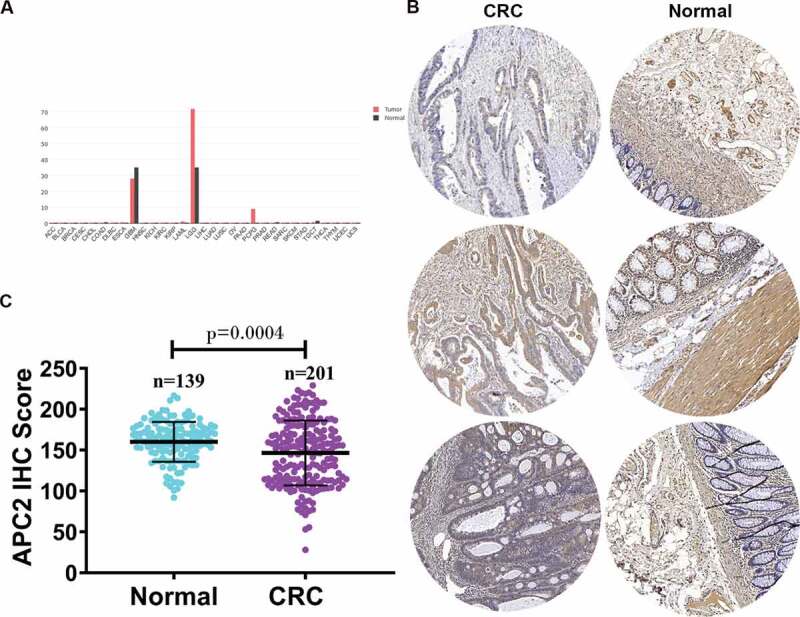
Relative expression of adenomatous polyposis coli 2 (APC2) in colorectal cancer (CRC) tumor tissues.

First, we analyzed the expression of APC2 mRNA in different cancer tissues using the Gene Expression Profiling Interactive Analysis database and revealed that APC2 expression was generally downregulated in most cancer tissues (Figure 1(a)). Subsequently, we determined the APC2 expression levels in 201 CRC tissues and 139 adjacent tissues using tissue microarray. The APC2 expression levels in three representatives CRC tissues and three representative adjacent normal tissues were shown in Figure 1(b). The CRC tissues were found to have lower APC2 expression level than the adjacent normal tissues (Figure 1(b)). Simultaneously, an IHC score was used to quantify the expression of APC2 detected by tissue microarray. IHC score assessment showed that the expression of APC2 in CRC was significantly lower than that in adjacent normal tissue (Figure 1(c)).

(a) The relative expression of APC2 in various cancers was analyzed using the Gene Expression Profiling Interactive Analysis database (http://gepia.cancer-pku.cn/detail.php?gene=apc2&tdsourcetag=s_pctim_aiomsg); (b) Relative expression of APC2 in three representative CRC tumor tissues (CRC) and three representative normal adjacent tissues (Normal) was evaluated using tissue microarray; (c) APC2 expression was represented by an immunohistochemistry (IHC) score determined by researchers.

### 3.2. APC2 is associated with better prognosis

**Figure 2.**
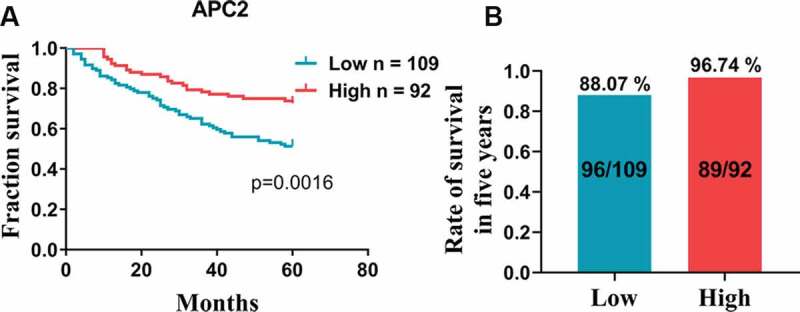
Survival analysis.

Based on the aforementioned results, we further analyzed the relationship between high and low expression levels of APC2 in CRC tissues and the patients’ survival periods. The survival analyses indicated that the 5-year survival of patients with high APC2 expression was higher than that of those low APC2 expression (Figure 2(a)). In addition, the survival of patients with high APC2 expression was 96.74% for 5 years, which was higher than that of patients with low APC2 expression (Figure 2(b)). Simultaneously, a total of 197 well-documented patients with CRC, consisting of 105 males and 92 females, were included in the clinical data analyses. The chi-square analysis showed that low APC2 expression was associated with lymphovascular invasion, lymph node metastasis, and TNM stage of CRC, indicating that low expression of APC2 was prevalent in patients who underwent late clinical analyses (Table 1).

**Table 1.** Correlations between APC2 expression and clinicopathological parameters in CRC patients.

Clinicopathological parameters	Total (*n* = 197)	APC2 expression^a^		*p* value
		Low (%)	High (%)	
Gender				
Male	105	55(52.38%)	50(47.62%)	0.524
Female	92	44(47.83%)	48(52.17%)	
Age (years)				
≤65	98	53(54.08%)	45(45.92%)	0.285
>65	99	46(46.46%)	53(53.54%)	
Lymphovascular invasion				
Negative	119	51(42.86%)	68(57.14%)	0.010*
Positive	78	48(61.54%)	30(38.46%)	
Depth of invasion				
T1-T2	9	5(55.56%)	4(44.44%)	1.000
T3-T4	188	94(50%)	94(50%)	
Lymph node metastasis				
N0	118	50(42.37%)	68(57.63%)	0.007**
N1-N2	79	49(62.03%)	30(37.97%)	
Distant metastasis				
M0	195	97(49.74%)	98(50.26%)	0.497
M1	2	2(100%)	0(0%)	
TNM stage				
I–II	118	50(42.37%)	68(57.63%)	0.007**
III–IV	79	49(62.03%)	30(37.97%)	
Pathological stage				
I–II	159	78(49.06%)	81(50.94%)	0.492
III–IV	38	21(55.26%)	17(44.74%)	

* *p* < 0.05; ** *p* < 0.01; ^a^ Using median expression level of APC2 as cutoff; APC2: adenomatous polyposis coli 2; CRC: colorectal cancer; TNM: tumor node metastasis.

Survival analysis showing the difference in (a) 5-year survival and (b) 5-year survival rate between adenomatous polyposis coli 2 (APC2) high and low expressing colorectal cancer (CRC) patients.

### 3.3. Clinical diagnostic value of APC2

**Figure 3.**
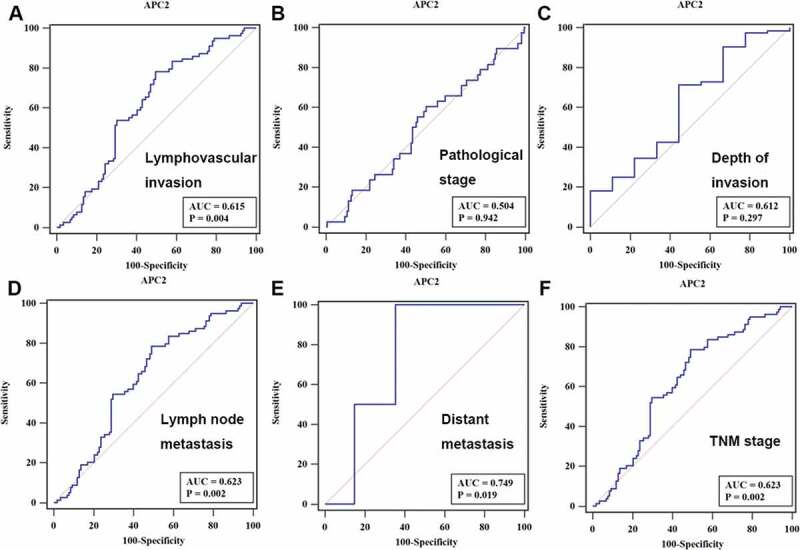
Receiver operating characteristic (ROC) curve analysis.

To analyze the potential role of APC2 in CRC diagnosis, ROC curves were developed by correlating the expression of APC2 with each patient’s diagnostic information. The ROC curve showed that the area under curve (AUC) values of APC2 for lymphovascular invasion, lymph node metastasis, and TNM staging were 0.615, 0.623, and 0.623 (p < 0.05), respectively (Figure 3).

The ROC curves of adenomatous polyposis coli 2 (APC2) that distinguished (a) lymphovascular invasion, (b) pathological stage, (c) depth of invasion, (d) lymph node metastasis, (e) distant metastasis, and (f) tumor node metastasis (TNM) staging.A.

## 4. Discussion

In the present study, we revealed that APC2 expression was lower in CRC tissue compared with adjacent normal tissue. Further, we divided CRC patients into two groups: high expression and low expression, to investigate the association between APC2 expression and several clinical parameters including lymphovascular invasion, pathological stage, depth of invasion, lymph node metastasis, distant metastasis, and TNM staging. Our findings showed that CRC patients in the lower APC2 expression group exhibited higher lymphatic invasion, lymph node metastasis, TNM staging, and a lower 5-year survival rate. In addition, the ROC curve revealed that APC2 may have potential diagnostic value with respect to distinguishing tumor lymphatic invasion, lymph node metastasis, and TNM staging.

APC2 is a homolog of APC. Although the tumor suppressive effect of APC has been established in a wide range of tumors, the function of APC2 has only been recognized in a limited number of tumors. For example, APC2 expression is decreased in breast tumors [18,19]. Inhibition of APC2 expression results in increased sensitivity of breast cancer cells to Panobinostat treatment [20]. In ovarian cancer, APC2 has been reported to be involved in miRNA-mediated tumor growth inhibition [21]. APC2 deficiency leads to susceptibility to granulosa cell tumor formation in mice and causes ovarian deficiency to promote tumor growth [22,23]. Previous studies have found that APC2 mRNA is downregulated in CRC tissue according to TCGA data [24]. In this study, tissue microarray results revealed that APC2 was downregulated in CRC, suggesting a functional role for APC2 protein expression in CRC tissues.

Furthermore, the development and metastasis of cancer is a multi-step process. Cancer cells of the primary tumor can migrate to regional lymph nodes and continue further to distant organs. It is believed that distal metastases originate as lymph node metastases, which are associated with a low 5-year survival rate in patients with cancer [25,26]. Moreover, lymphovascular invasion is considered an independent prognostic factor that can affect clinical decision-making in administering adjuvant chemotherapy to patients with stage II CRC [27]. Moreover, the basis of the TNM staging system is closely related to the metastasis of lymph nodes. Therefore, evaluation of CRC metastasis is of great significance for tumor prognosis. In this study, we revealed that low expression of APC2 was associated with lymphatic vessel infiltration, lymph node metastasis, and high TNM stage. This result was consistent with that of previous studies showing that APC2 is associated with lymphangiogenesis and lymph node metastasis [28].

In addition, APC2 plays an important role in regulating the cytoskeleton [29,30]. Low expression of APC2 may result in an abnormal cytoskeleton leading to increased cell motility, enhanced cell migration and invasion. Although there is currently no evidence indicating that APC2 regulates CRC cell behavior, we can hypothesize that the cytoskeletal regulation function of APC2 in CRC patients may contribute to the development of malignant cancer, such as lymphatic invasion and lymph node metastasis. In this study, corresponding to the high metastatic frequency of CRC in patients with low APC2 expression, we also found that the 5-year survival rate of these patients was significantly lower than that of patients with high APC2 expression, suggesting that low APC2 expression predicted poor prognosis of CRC. This result is consistent with previous studies indicating that APC2 is associated with lymphangiogenesis and lymph node metastasis in lung cancer [28].

In general, we found that APC2 expression was associated with the depth of invasion, lymph node involvement, and TNM staging. Our study also indicated that low APC2 expression in patients with CRC was associated with poor tumor prognosis, suggesting that APC2 could be a useful diagnostic and prognostic marker for CRC patients.

## Research highlights

1. APC2 was found to be a potential biomarker for colorectal cancer.

2. APC2 was downregulated in colorectal cancer tissue.

3. APC2 was associated with lymphovascular invasion, lymph node metastasis, and TNM staging.
